# Supplementary material for: PyUUL provides an interface between biological structures and deep learning algorithms
Source: Nat Commun. 2022 Feb 18;13:961. doi: 10.1038/s41467-022-28327-3 (PMC8857184; doi:10.1038/s41467-022-28327-3)
Supplement: Supplementary file 1 — Supplementary Information [file 41467_2022_28327_MOESM1_ESM.pdf]

PyUUL provides an interface between biological structures and deep learning algorithms

Orlando et al.

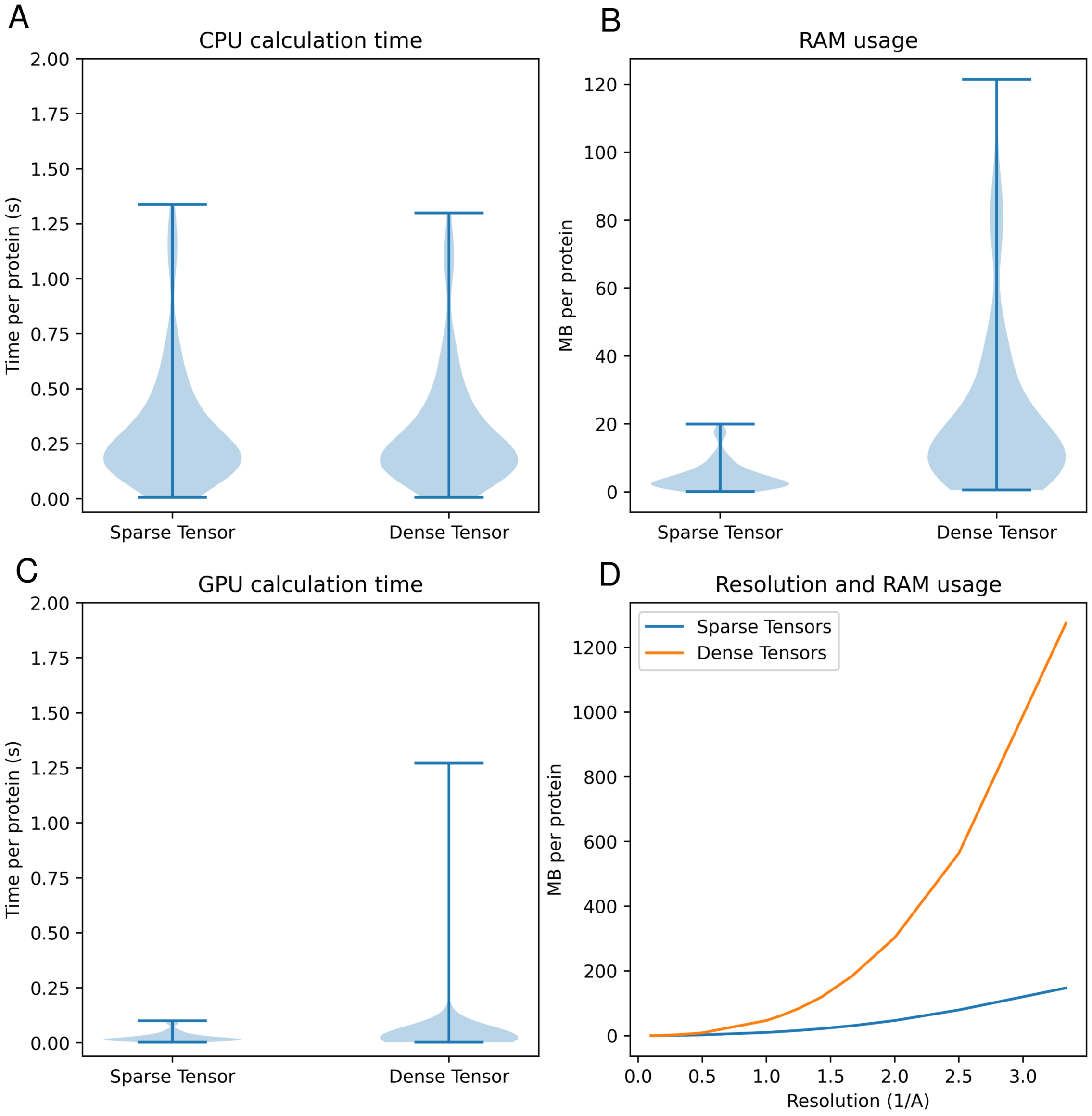

Supplementary Figure 1: **Computational performances of the voxel representation using different settings.** ***A*** computational time required to calculate the voxel-based representation of proteins using sparse and dense tensors on CPU with an inverse resolution of 1 $\mathring{A}$. ***B*** Size of the final tensors using sparse and dense tensor types respectively. ***C*** Computational time required to calculate the voxel-based representation of proteins using sparse and dense tensors on GPU with an inverse resolution of 1 $\mathring{A}$. ***D*** relationship between size of the final voxel-based protein representation and resolution. Source data are provided as a Source Data file.

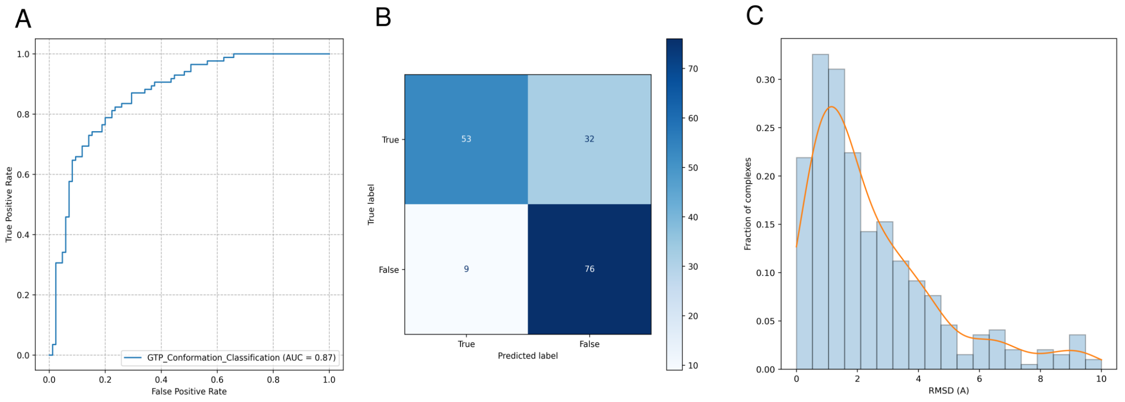

Supplementary Figure 2: **performances of classification and optimization of protein-GTP complexes** The figure shows the ROC curve and the confusion matrix of the classification task of the protein-GTP complex optimization. The network is trained to recognize complexes in which GTP has been randomly rotated from the original ones. ***A*** **:** ROC curve of the classification task of the GTP conformation optimization. The ROC curve shows the performances of the neural network in discriminating the rotated protein-GTP complex and the original ones. ***B*** **:** confusion matrix of the classification task of the GTP conformation optimization. ***C*** **:** Distribution of the RMSD of the optimized GTP-protein conformation respect to the crystal structure one. Only the 473 complexes (out of 515) that converged to a solution are shown in the plot. Source data are provided as a Source Data file.

Supplementary Table 1: **Pearson's correlation coefficient between between GTP pocket signature and pocket point cloud distance matrices:**

Number Of Features	PCC
10	0.704057118175314
9	0.718278258159284
8	0.7088703967154719
7	0.6961463833666499
6	0.6785070274424595
5	0.6311800942811507
4	0.6236681068585134
3	0.580430972143755
2	0.5857434310054538

The table shows the drop of performances for the encoding of the GTP binding pocket with the decrease of the number of dimensions of the encoding. The lower the number of dimensions of the encoding is, the less space the network has to compress structural information. Source data are provided as a Source Data file.

Supplementary Notes

1 Network architectures

The network for Alpha helix identification is made of two branches. The first one is a three layer 3D convolutional neural network with kernel of 7, padding of 3 and 10 output

channels. Between every layer we applied dropout of 0.1, instance normalization and a Leaky ReLU activation. The last convolutional layer has no dropout and no instance normalization, along with 100 output channels. The output of this network has a tensor of the same size of the input tensor, but with 100 channels only. The second branch is a 3 layers feed forward neural network and it is applied on the channels dimension of the output tensor of the 3d convolutional branch. The first two layers have 100 neurons each and Leaky ReLU activation, while the last one consists in a single neuron with sigmoid activation. The network has been trained with Adam optimizer [1] from pytorch [2] with default parameters.

For what concerns the Network for gradient-based protein structure optimization, the spatial transformer is in charge of learning the transformation matrix in order to transform the input data. Spatial transformer is defined with a self-attention network. Attention and prediction branches are, as usual, symmetric, but the attention has a final softmax activation performed on the voxels dimension. Both branches are made but a single convolutional 3D layer with kernel of 5, stride of 2 and padding of 2, followed by max pooling and instance normalization. The transformation matrix is obtained performing an affine transformation the dot product of the prediction and attention output using pytorch `affine_grid` and `affine_sampling` functions, as described in [3]. The result of this operation is a (batch,3,4)-shaped tensor that represent the transformation matrix and it is used to transform the input data. The next part of the network is again made of two symmetric (attention and prediction) branches. Each one is made of two 3D convolutional layers with kernel of 5 and padding of 2, each one followed by dropout of 0.1, instance normalization and hyperbolic tangent activation. As above, attention has a final softmax activation on the voxels dimension and the final output of the network is the dot product of the two branches. This output is than used as input for a 3 layers feed forward neural network. Each layer has 40 hidden neurons and it is followed by layer normalization, dropout of 0.1 and ReLU activation. The output layer has a single neuron as output and sigmoid activation.

2 Channel hashing

The voxel representation, like it happens for RGB digital pictures, have different channels. Every channel contains information about a different type of atom, meaning that each channel is independent from the others. The user can define how to regroup atoms based on its need (see PyUUL documentation for a description of the procedure to get custom channel hashing). As a default, PyUUL uses the hashing proposed in Torch Protein Library [4], with the addition of independent channels for the backbone atoms, for a total of 16 channels for proteins. Alternatively, PyUUL also provides a simpler encoding, in which the channel to which an atom belongs is only based on the chemical element (C = 0, N = 1, O = 2, everything else = 3).

3 Network performances discussion

In this section we will discuss in more detail the performances and results of the networks we presented in the main article. These perfomances have the purpose to show that the volumetric representation of biological molecules can be used to feed neural networks and that they can learn, within a certain degree, general rules. These performances are

therefore not meant to be used as a comparison to state of the art methods, but just as a proof of concept that common computer vision ML techniques can be applied to biological problems using PyUUL. The development of an actual bioinformatics tool, such as a docking algorithm, requires ad-hoc network architectures, able to effectively deal with the problem under scrutiny. The evaluation of the End-to-end structure-based protein clustering network as been performed on 491 proteins belonging to 4 different SCOP classes. In order to estimate the capability of the network to generalize structural information, we split the dataset in a train (393 proteins) and a test (98 proteins). With these settings, the network is able to converge and provide a protein signature that conserves the clustering provided by SCOP. From our tests, however, we observed that the network experiences convergence issues when increasing the number of classes. This is most probably due to the fact that proteins belonging to the same SCOP class often have only partial coverage, increasing the intra-cluster heterogeneity of the structures. This can most probably be addressed with an ad-hoc architecture that emphasizes a partial matching of two structures (i.e. zooming and cropping transformations of the spatial transformer). The effect of different loss functions, such as triplet loss [5], might be also be explored. The development of such an architecture is, however, out of the scope of this paper.

For what concerns the gradient-based protein structure optimization, Figure 2 shows the performances of the GTP optimization problem described in the main article. The problem has been addressed in two steps: a first part in which the protein learns to classify GTP-protein complexes in which the ligand has been randomly rotated (negative labels) from the original ones (positive labels) and a second one in which the network docks the ligand into the binding pocket. We decided to encode the first step as a classification problem for simplicity, so that we could have used binary-crossentropy as loss. Other possible approaches would be, for instance, to let the network learn the magnitude of the rotation applied to the ligand (where the original pose has a rotation of 0 degrees), performing therefore a regression. We first wanted to assess if the classification problem alone was correctly addressed by the network: we therefore performed a stratified 5 folds cross-validation on the classification problem alone in which proteins with higher sequence identity than 30% are grouped together. The network reaches an area under the ROC curve (AUC) of 0.87. The ROC curve, along with the confusion matrix obtained applying a binarization (threshold of 0.2) to the predictions, is shown in Figure 2A and 2B. In order to validate the whole optimization process, we ran another 5-folds cross-validation: for every split, we trained the network on the aforementioned classification problem using the data of the training set and we used the trained network to reposition the ligands of the complexes of the testing set. In order to deal with numerical problems connected to gradient vanishing (i.e. when GTP is too far from the optimal position), as a very first operation of the optimization step we randomly rotate and translate 10 times the input GTP structure. In other words, we sample several initial conformations of the GTP in order to increase the chances the network will converge. We then optimize their poses and we keep the one to which the network give the highest confidence as final result. Figure 2C shows the distribution of the RMSD between the optimized GTP and the original one. 313 out of 515 of the optimized complexes had a root-mean-square deviation of atomic positions (RMSD) lower than 2 with respect to the original crystal structure, 68 between 2 and 3, 93 between 3 and 10, and 42 did not converge to a solution after 100 iterations. Most of the times this pipeline manages to find solutions that are very close to the true ones. However the fact that 42 complexes did not converge to

any solution suggests that a docking belonging to this class of algorithm might need an ad-hoc optimizer that explores the solution space in a more efficient way, in particular if flexible docking, in which dihedral angles of the ligand are used as additional parameters to optimize, wants to be addressed. For what concerns the possibility to define a protein signature encoding with point clouds, we evaluated the capability of a foldNet to grasp structural information of GTP binding pockets used in the docking example. We validated the network performing a stratified 5-folds cross-validation, where proteins with higher sequence identity of 30% are grouped together. The validation and training was performed comparing the distance matrix between the signatures of the binding pockets with the distance between the point cloud. The distance between two cloud points is defined as the RMSD between the matching point identified with a fast global registration algorithm [6]. The network converges quickly (withing 30 epochs) to a pearson's correlation coefficient of 0.72 using 10 encoding features, showing it can learn the structural information of the pocket. The algorithm we are using is basically a compression algorithm and another interesting result is that, as expected, the correlation decreases reducing the number of encoding dimensions (Supplementary Table 1). In other words, the network has less room to store structural information and therefore the loss of information is more pronounced.

Supplementary references

[1] Kingma, D. P. & Ba, J. Adam: A method for stochastic optimization. *arXiv preprint arXiv:1412.6980* (2014).

[2] Paszke, A. *et al.* Automatic differentiation in pytorch (2017).

[3] Jaderberg, M., Simonyan, K., Zisserman, A. & Kavukcuoglu, K. Spatial transformer networks. *arXiv preprint arXiv:1506.02025* (2015).

[4] Derevyanko, G. & Lamoureux, G. Torchproteinlibrary: A computationally efficient, differentiable representation of protein structure. *CoRR* **abs/1812.01108** (2018). URL `http://arxiv.org/abs/1812.01108`. `1812.01108`.

[5] Chechik, G., Sharma, V., Shalit, U. & Bengio, S. Large scale online learning of image similarity through ranking. *Journal of Machine Learning Research* **11** (2010).

[6] Zhou, Q.-Y., Park, J. & Koltun, V. Fast global registration. In *European conference on computer vision*, 766–782 (Springer, 2016).
