# Supplementary material for: PyUUL provides an interface between biological structures and deep learning algorithms
Source: Nat Commun. 2022 Feb 18;13:961. doi: 10.1038/s41467-022-28327-3 (PMC8857184; doi:10.1038/s41467-022-28327-3)
Supplement: Supplementary file 3 — Description of Additional Supplementary Files [file 41467_2022_28327_MOESM3_ESM.pdf]

Description of Additional Supplementary Files

File Name: Supplementary Movie 1
Description: The evolution of the neural network for alpha helices identification during training. It refers to figure 2A

File Name: Supplementary Movie 2
Description: The evolution of the neural network for protein signature clustering during training. It refers to figure 2B

File Name: Supplementary Movie 3
Description: Pose optimization of GTP using a pretrained neural network. It refers to figure 2C
