# Supplementary material for: PyUUL provides an interface between biological structures and deep learning algorithms
Source: Nat Commun. 2022 Feb 18;13:961. doi: 10.1038/s41467-022-28327-3 (PMC8857184; doi:10.1038/s41467-022-28327-3)
Supplement: Supplementary file 7 — Reporting Summary [file 41467_2022_28327_MOESM7_ESM.pdf]

nature portfolio

Corresponding author(s): Gabriele Orlando

Last updated by author(s): Dec 21, 2021

Reporting Summary

Nature Portfolio wishes to improve the reproducibility of the work that we publish. This form provides structure for consistency and transparency in reporting. For further information on Nature Portfolio policies, see our Editorial Policies and the Editorial Policy Checklist.

Statistics

For all statistical analyses, confirm that the following items are present in the figure legend, table legend, main text, or Methods section.

n/a	Confirmed	
☐	☒	The exact sample size (*n*) for each experimental group/condition, given as a discrete number and unit of measurement
☒	☐	A statement on whether measurements were taken from distinct samples or whether the same sample was measured repeatedly
☐	☒	The statistical test(s) used AND whether they are one- or two-sided<br>*Only common tests should be described solely by name; describe more complex techniques in the Methods section.*
☒	☐	A description of all covariates tested
☐	☒	A description of any assumptions or corrections, such as tests of normality and adjustment for multiple comparisons
☐	☒	A full description of the statistical parameters including central tendency (e.g. means) or other basic estimates (e.g. regression coefficient) AND variation (e.g. standard deviation) or associated estimates of uncertainty (e.g. confidence intervals)
☒	☐	For null hypothesis testing, the test statistic (e.g. *F*, *t*, *r*) with confidence intervals, effect sizes, degrees of freedom and *P* value noted<br>*Give P values as exact values whenever suitable.*
☒	☐	For Bayesian analysis, information on the choice of priors and Markov chain Monte Carlo settings
☒	☐	For hierarchical and complex designs, identification of the appropriate level for tests and full reporting of outcomes
☐	☒	Estimates of effect sizes (e.g. Cohen's *d*, Pearson's *r*), indicating how they were calculated

Our web collection on statistics for biologists contains articles on many of the points above.

Software and code

Policy information about availability of computer code

Data collection	The PDBs have been collected using PDB's batching script available at PDB website that allows the interaction with the web API. A pymol (version 2.5.2) script has been used to modify the PDB structures and to build the structure figures
Data analysis	The library is written in python 3.8. As additional packages it uses numpy 1.19, pytorch 1.9 and scikit-learn 1.0.1. All of these are open sources packages. The output of this work is PyUUL. The library is open source and available at https://bitbucket.org/grogdrinker/pyuul/

For manuscripts utilizing custom algorithms or software that are central to the research but not yet described in published literature, software must be made available to editors and reviewers. We strongly encourage code deposition in a community repository (e.g. GitHub). See the Nature Portfolio guidelines for submitting code & software for further information.

Data

Policy information about availability of data

All manuscripts must include a data availability statement. This statement should provide the following information, where applicable:

- Accession codes, unique identifiers, or web links for publicly available datasets
- A description of any restrictions on data availability
- For clinical datasets or third party data, please ensure that the statement adheres to our policy

All the code and the PDB structures used in this study are available at https://bitbucket.org/grogdrinker/pyuul/. All the structures have been downloaded from the Protein Data Bank (PDB)

Field-specific reporting

Please select the one below that is the best fit for your research. If you are not sure, read the appropriate sections before making your selection.

- [x] Life sciences
- [ ] Behavioural & social sciences
- [ ] Ecological, evolutionary & environmental sciences

For a reference copy of the document with all sections, see nature.com/documents/nr-reporting-summary-flat.pdf

Life sciences study design

All studies must disclose on these points even when the disclosure is negative.

Sample size	We used all the structures we could select using the standard filters from PDB website
Data exclusions	We did not perform any data esclusion
Replication	We provide data and tutorials to replicate our findings
Randomization	We perform grouped 5-folds cross validation when we test our networks. Proteins with sequence identity greater than 30% are always grouped together in the same fold
Blinding	We present a python library to deal with biological structures, we therefore do not generate new data. Moreover, the neural networks we use are not meant to solve biological problems, but to show potential uses of the library in machine learning and the convergence of simple networks on tensorial representation of proteins. Blinding is therefore not relevant

Reporting for specific materials, systems and methods

We require information from authors about some types of materials, experimental systems and methods used in many studies. Here, indicate whether each material, system or method listed is relevant to your study. If you are not sure if a list item applies to your research, read the appropriate section before selecting a response.

Materials & experimental systems

n/a	Involved in the study	
x		Antibodies
x		Eukaryotic cell lines
x		Palaeontology and archaeology
x		Animals and other organisms
x		Human research participants
x		Clinical data
x		Dual use research of concern

Methods

n/a	Involved in the study	
x		ChIP-seq
x		Flow cytometry
x		MRI-based neuroimaging
